# Relative significances of pH and substrate starch level to roles of *Streptococcus bovis* S1 in rumen acidosis

**DOI:** 10.1186/s13568-016-0248-2

**Published:** 2016-09-22

**Authors:** Lianmin Chen, Shimin Liu, Hongrong Wang, Mengzhi Wang, Lihuai Yu

**Affiliations:** 1 Laboratory of Metabolic Manipulation of Herbivorous Animal Nutrition, College of Animal Science and Technology, Yangzhou University, Yangzhou, 225009 China; 2 School of Animal Biology, The University of Western Australia, Crawley, WA 6009 Australia

**Keywords:** Rumen acidosis, Lactate, *Streptococcus bovis*, Starch, Rumen pH

## Abstract

To clarify the relative importance of pH and substrate starch level in fermentation characteristics and regulatory mechanism of *Streptococcus bovis* S1 in rumen acidosis, an in vitro fermentation of three levels of soluble starch (1, 3 and 9 g/L) was established with pH in the media were maintained constant at 5.5 or 6.5. The results showed that the dominant product of *S. bovis* S1 was lactate at both pH, the production depended on the starch level, and more lactate was produced at pH 6.5 than that at pH 5.5 (*P* < 0.001). At pH 5.5, the activity of lactate dehydrogenase (LDH) and *α*-amylase (α-AMY), their abundances, the relative expressions of *LDH*, *PFL* (gene encoding pyruvate formate-lyase), *CCPA* (gene encoding global catabolite control protein A) and *α*-*AMY* genes were higher than those at pH 6.5 (*P* < 0.05), whereas the concentration of fructose-1,6-diphosphate (FDP) was lower. The activity of LDH, α-AMY and FDP, and the relative expressions of *LDH*, *PFL, CCPA* and *α*-*AMY* genes were, in general, positively related to the starch level. The canonical regression analysis indicated that the pH had more profound effect compared with the starch level, in terms of the acid productions, enzyme activity and gene expressions. It was concluded that the fermentation of *S. bovis* was regulated at the transcription level in response to both pH and substrate starch concentration, but more sensitive to pH changes.

## Introduction

Rumen acidosis is a common digestive disorder in ruminants fed high concentrate diets, and typically characterized with low rumen pH that results in changes in rumen fermentation and microbial profiles, disturbed intake and reduced productive performances (Tajima et al. 2001; Rotger et al. 2006). However, the fate of the resultant effects in the rumen is unclear. In normal conditions, the pH of the rumen fluid is about 6.5 (Wang et al. 2015). When a large amount of starch is applied to the diet, ruminal pH decreases below 5.5 with an accumulation of volatile fatty acids (VFA) and lactate, leading to an occurrence of rumen acidosis (Kleen and Cannizzo 2012). These two events are usually simultaneous, and therefore, confounded (Calsamiglia et al. 2012). There exists an issue of whether acidosis is attributed to the reduction of pH or to over-supplied non-structural carbohydrate (NFC) in the diet. A distinction of the roles of either events is essential, as the sequence is pH dependent. Then the term acidosis and the use of buffers and alkalizers is justified; whereas if the outcome is due to the excess of NFC in the diet, then the use of buffers would have a limited effect, and we should be looking for pertinent solutions and accurate terminology (Calsamiglia et al. 2012).

Our previous work with dairy cows demonstrated that rumen acidosis was associated with an initially over growth of *Streptococcus bovis* (*S. bovis*) and the inability of lactate utilizing bacteria like *Megasphaera elsdenii* and *Selenomonas ruminantium* in the rumen (Wang et al. 2015). In the past decades, the fermentative capacity of *S. bovis* has been studied and suggested that the pH in the rumen fluid was an important indicator in inducing organic acid fermentation pathway shift. *S. bovis* produces lactate mainly when pH is lower than 5.5, however it shifts to formate, acetate and ethanol fermentation when pH is higher than 6.0 (Gunsalus and Niven 1942; Russell and Hino 1985; Asanuma and Hino 1997). To some extent, this might explain why a little lactate is detected when ruminants suffer from subacute rumen acidosis while in cases of acute rumen acidosis, a large amount of lactate is accumulated.

Recent studies have shown that when a readily fermented energy source such as glucose is used as a sole carbon source, lactate is produced, and more lactate is produced as a result of an excess supply of glucose (Asanuma and Hino 2000; Asanuma et al. 2008, 2009). It seems that both pH and the fermentation substrate abundance play important roles in regulating *S. bovis*’ lactate production, however, to our knowledge few studies have been conducted to directly evaluate which factor is more effective. In this study we used starch, a most abundant substrate in diet for ruminants, as the source of readily fermentable carbohydrate source in an in vitro model, and investigated its utilization by *S. bovis* in a condition that the pH of the culture media was unchanged.

## Materials and methods

### Strain and seed culture

A new *S. bovis* strain S1 (CCTCC AB 2016240) isolated by Chen et al. (2016) was used in this study. The seed culture was carried out in a modified MRS medium. The media contained (g/L): tryptone 10.0, yeast extract 5.0, beef extract 10.0, glucose 10.0, K_2_HPO_4_ 2.0, Tween 80 1.0, MgSO_4_∙7H_2_O 0.2, MgSO_4_∙H_2_O 0.05, ammonium citrate 2.0, C_2_H_3_NaO_2_ 5.0. All media were sterilized by autoclaving at 121 °C and 15 psi for 15 min. The culture was carried out in an anaerobic workstation (DG250, Don Whitley Scientific, England) at 37 °C.

### Experimental treatments, incubation and sampling

The strain S1 was cultured in 250 mL serum bottles containing 200 mL basal medium. The basal medium (Asanuma et al. 2009) contained in 1 L: 1.0 g tryptone, 1.0 g yeast extract, 0.6 g cysteine-HCl, 0.45 g K_2_HPO_4_, 0.45 g KH_2_PO_4_, 0.9 g NaCl, 0.12 g CaCl∙2H_2_O, 0.19 g MgSO_4_∙7H_2_O, 0.9 g (NH_4_)_2_SO_4_. The culture bottles were incubated at 37 °C in a horizontal position in an anaerobic shaking water bath at a shaking speed of 160 rev/min.

The experimental treatments were a 3 × 2 factorial design: three levels of soluble starch (Sigma, PN. S9765) as the sole carbohydrate source in the media: 1 g/L, 3 g/L (close to a normal range in the rumen fluid) and 9 g/L (excessive level) respectively, while the pH of the media was maintained constantly at 5.5 (acidosis) or 6.5 (normal) by using 10 % NaOH (wt/vol) or 10 % HCl (vol/vol). The pH of the media was monitored continuously using a pH meter (Seven Excellence equipped with InLab Routine Pro-ISM meter, Mettler-Toledo, Switzerland), and adjusted to 5.5 or 6.5 accordingly by infusing the alkali or the acid with a peristaltic pump (LabV1, Baoding Shenchen Precision Pump Co., Ltd. China). There were triplicates for each treatment. For the culture, 2.0 mL of the seed culture was used as the inoculator when the culture attained the optical density (OD) of 0.4 (exponential phase). Cell growth was monitored by measuring OD values at 600 nm by using SpectraMax M5 plate reader (Molecular Devices Corporation, USA). The medium fluid was respectively collected at 13 h (during the exponential phase) and 40 h (during the plateau phase) for analyses of fermentation products, bacterial enumeration, enzyme activity and gene expression.

### Bacterial enumeration, fermentation product determination and enzyme activity assays

For bacterial enumeration, a drop plate method was used according to Chen et al. (2003) with MRS agar plates and incubating at 37 °C for 24 h. In order to break the cell wall, bacterial cell homogenization was performed by mixing 1 mL bacterial fluid with 0.3 g of zirconia-silica beads (0.1 mm in diameter), and then homogenized in a FastPrep-24 Automated system (MP Biomedicals, Solon, OH, USA) for 2 min, followed by sonication for 2 min (100w, 20 cycle) by using a VCX-130 Sonicator (Sonics, USA) in an ice bath. Cell debris was removed by centrifugation at 8000 rpm for 10 min at 4 °C, and the supernatant was collected for further analysis; A high-performance liquid chromatographer (HPLC, Agilent 1200, USA) equipped with an acclaim OA column (PN. 062902, Thermo, USA) and a UV detector was used to detect organic acid (formate, acetate, and lactate) concentrations in the supernatant. The column temperature was kept at 30 °C, and the mobile phase was 100 mM Na_2_SO_4_ (pH 2.65, adjust with methane sulfonic acid), and its flow rate set at 0.6 mL/min. Organic acids were then measured by determining the UV 210 nm.

The supernatant was also used for measuring the activity of intracellular enzymes α-Amy and LDH, and FDP concentration. In brief, FDP concentration was measured by an enzymatic procedure of Racker (Bergmeyer 2012) using a commercial JSAY139 FDP kit (Jie Shi Kang Biotech Co., Ltd. China). LDH was detected by a method similar to De Vries et al. (1970) using a commercial kit (PN. A020, Jiancheng Biotech Co., Ltd. China). α-Amy activity was measured by an iodine–starch method (Gil’manov et al. 1981) using a C016-1 kit (Jiancheng Biotech Co., Ltd. China). The total sugar concentration in the media was assayed by a colorimetric method (Dubois et al. 1956) using a A145 kit (Jiancheng Biotech Co., Ltd. China), and then converted into the amount of soluble starch consumed by multiplying a factor of 1.11 (1 unit soluble starch forms 1.11 unit glucose).

### Analysis of gene transcription

Before RNA extracting, a volume of 250 μL lysozyme solution (20 mg/mL) was add to 1 mL bacterial fluid and incubated at 37 °C for 8 min to breakdown the cell walls. Total RNA was then extracted by using a RN43-EASYspin Plus Kit (Aidlab Biotech Co., Ltd. China), and the extraction was carried out according to the manufacturer’s protocol. RNA quality was assessed by using Agilent 2100 Bioanalyzer (Agilent Technologies, Palo Alto, CA, USA). The RNA was reverse-transcribed to cDNA with random-hexamer primers and Quant reverse transcriptase (Tiangen, Biotech Co., Ltd. China). The cDNA was amplified by real-time PCR with Power SYBRR Green PCR Master Mix and Power SYBR Green RT-PCR Reagents Kit (FP205, TIANGEN, Beijing). The RT-qPCR was performed in an ABI real-Time PCR (ABI 7500), operated according to manufacturer’s instructions. The reaction was first performed in a 20 μL reaction solution containing 10 μL 2 × SuperReal PreMix Plus, 1.6 μL primer, 1 μL cDNA, 0.4 μL 50 × ROX and 7.0 μL Rnase-free water. After a single pre-denaturation cycle at 95 °C for 15 min, the amplification was performed for 45 cycles (95 °C for 10 s and 60 °C for 32 s). The *16S* rRNA was used as an internal reference gene, and the primer information was shown in Table 1. The PCR products were electrophoresed on a 1 % agarose gel and visualized upon staining with ethidium bromide to detect the specificity of polymerase chain reaction. Gene expression was calculated by using the 2^−ΔΔCt^ method according to (Pfaffl 2001), and PCR amplification efficiency was calculated according to (Ramakers et al. 2003).

**Table 1:** Oligonucleotide primers used for RT-qPCR techniques

Items	Sequence of primers (5′-3′)	Reference	GeneBank ID	Production	Amplification efficiency (%)
*16S*	F:GAACACCGGTGGCGA	(Asanuma et al. 2010)			97.13
	R:CTCATCGTTTACGGCG				
*PFL*	F:GGTTACATCTACGACTACGA	This study	AB014686.1	119	98.54
	R:TGGCTACGAAGACGAGTA				
*LDH*	F:GGTTCTTCTTACGCATTCG	This study	U60997.1	190	99.67
	R:TAACTACAAGGTCAGCATCT				
*α*-*AMY*	F:TCAAGCACTGGAATCAACTA	This study	U04956.1	109	101.12
	R:GCCGTAATAATCTCCGTAGA				
*CCPA*	F:CCGTTGGTGTTGTTATTCC	This study	AB028599.3	126	95.24
	R:TATCGTCGTCTTCATCACTT				

### Statistical analysis

Results were expressed as the least square mean ± standard error of means (SEM), and the statistical analysis was performed using SPSS 20.0 (IBM, USA). The general linear model procedure for repeated measures were used to examine the fixed effects of pH (2 levels), soluble starch concentrations (3 levels) and their interactions. The multiple comparisons were performed via the Duncan’s test. To distinguish relative effects of pH and starch concentration, the original measure was calculated for their corresponding standard score “*Z*”, *Z* = (*x* *−* *μ*)/*σ*, where *μ* is the mean of all the measures, *σ* is the standard deviation of all the measures. Those derived *Z* scores were then used in a binary linear regression model: *Y* = *a* + *b*_*pH*_X_pH_ + *b*_*C*_X_C_, where *Y* is the dependent variable, *a* is the constant term, X_pH_ and X_C_ are the independent variables, *b*_*pH*_ and *b*_*C*_ are the regression coefficients. In addition, a linear regression was conducted to analyze the growth rate of the strain S1 during the log phase. The model is as follows: *Y* = *kX* + *b*, where *Y* is the OD reading, *X* is hour of the incubation, *k* is the steepness of the curve (i.e., growth rate, OD unit/h), and *b* is the constant term. *P* values of less than 0.05 (*P* < 0.05) were declared to be significant.

## Results

### Cell growth

**Fig. 1:**
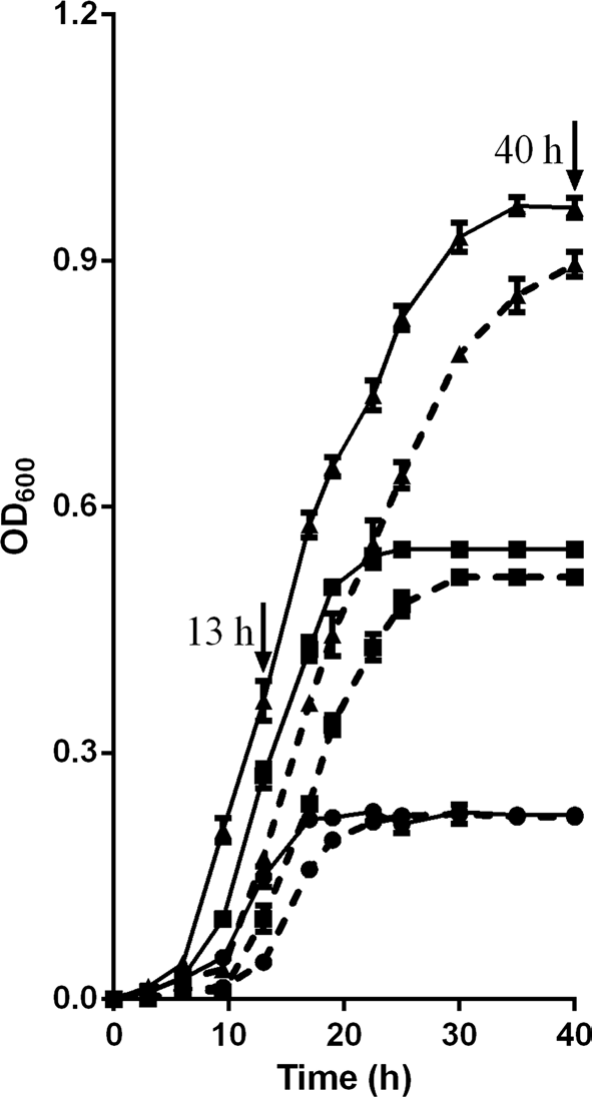
Effects of soluble starch concentrations and pH on growth of *S. bovis* S1. Soluble starch concentrations are 1 g/L (●), 3 g/L (■) and 9 g/L (▲); *Solid* and *broken lines* represent the pH at 6.5 and 5.5. Values are means (n = 3) with their standard errors represented by *vertical bars*

Growth curves of the bacteria with incubation time are shown in Fig. 1. At pH 6.5, the optical density reached 0.22, 0.54 and 0.93 at 17, 22.5, and 30 h of the late exponential phase respectively for starch levels of 1, 3 and 9 g/L; whereas at pH 5.5, the optical densities were 0.22, 0.50 and 0.90 at 25, 30 and 40 h of the late exponential phase. At 13 h and 40 h, the bacteria on all six treatments reached the exponential growth phase or the plateau phase. The growth rates (i.e., the *k* values) of strain S1 during the log phase are shown in Table 2. The *k* value increased, so the mean generation time decreased with an increasing dose of starch or at the higher pH.

**Table 2:** Linear regression analysis of the growth rate (*k*, mean and standard error, SEM_k_) of *S bovis S1*during the exponential phase in media

PH	Concentration (g/L)	*K* ^a^	SEM_*k*_	R_adjust_	*P*_R_	MGT (h)^b^
6.5	1	0.020	0.001	0.993	<0.001	50.00
	3	0.038	0.002	0.966	<0.001	26.32
	9	0.044	0.002	0.979	<0.001	22.73
5.5	1	0.018	0.002	0.893	<0.001	55.56
	3	0.025	0.002	0.900	<0.001	40.00
	9	0.027	0.001	0.937	<0.001	37.04

^a^The slope (i.e., growth rate) of the growth curve during the log phase

^b^Mean generation time, 1/*k*

### Fermentation products

The final concentration of lactate, the total organic acids (formate, acetate and lactate) and the total organic acid production per gram soluble starch consumed in the media are shown in Table 3. Lactate was the dominant product by *S. bovis*, and its quantity increased in a dose-dependent manner, accounting for 71 % up to 95 % of the total organic acids, and was higher at pH 6.5 than pH 5.5 (*P* < 0.05). A small amount of formate was produced as well, and its quantity accounted for lesser than 4 % of the total organic acids, depending on the starch level. There was an interaction between pH and starch level on the formate production: decreasing at pH 6.5 but increasing at pH 5.5, with the starch levels (*P* < 0.05). The amount of acetate produced declines with the starch level (*P* < 0.05), and did not differ between pH 5.5 and pH 6.5.

**Table 3:** Effects of soluble starch concentrations and pH on organic acids production

Items	PH	Concentration (g/L)			Mean_pH_	SEM_Total_	*P*_pH_	*P*_C_	*P*_pH×C_
		1	3	9					
Formate (mM)^1^	6.5	1.25	1.14	0.58	0.99	0.843	0.077	0.135	<0.001
	5.5	0.48	0.92	1.21	0.87				
	Mean_C_	0.87	1.03	0.90					
Acetate (mM)^1^	6.5	4.15	3.83	2.05	3.34	0.210	0.701	<0.001	0.093
	5.5	3.57	4.25	2.00	3.28				
	Mean_C_	3.86^a^	4.04^a^	2.03^b^					
Lactate (mM)1	6.5	14.66	34.28	56.01	34.98^a^	1.950	0.001	<0.001	0.641
	5.5	9.83	26.62	47.63	28.03^b^				
	Mean_C_	12.25^c^	30.45^b^	51.82^a^					
OA_total_ (mM)^2^	6.5	20.07	39.24	58.65	39.32^a^	2.114	0.001	<0.001	0.962
	5.5	13.85	31.80	50.85	32.16^b^				
	Mean_C_	16.96^c^	35.52^b^	54.75^a^					
OA_per_ (mM)^3^	6.5	20.07	13.08	8.38	13.84^a^	0.591	<0.001	<0.001	<0.001
	5.5	13.85	10.60	10.17	11.54^b^				
	Mean_C_	16.96^a^	11.84^b^	9.27^c^					
Formate (%)^4^	6.5	6.26	2.87	1.00	3.38^a^	0.175	0.003	<0.001	<0.001
	5.5	3.23	2.92	2.37	2.84^b^				
	Mean_C_	4.74^a^	2.90^b^	1.68^c^					
Acetate (%)^4^	6.5	20.63	9.82	3.50	11.32^b^	1.031	0.003	<0.001	0.102
	5.5	25.83	13.42	3.93	14.39^a^				
	Mean_C_	23.23^a^	11.62^b^	3.72^c^					
Lactate (%)^4^	6.5	73.12	87.31	95.50	85.31^a^	0.929	0.006	<0.001	0.586
	5.5	70.94	83.66	93.70	82.77^b^				
	Mean_C_	72.03^c^	85.48^b^	94.60^a^					

^1^Organic acids concentration at 40 h

^2^Total organic acids (formate, acetate, and lactate) concentration at 40 h

^3^Total organic acids production per gram soluble starch at 40 h

^4^Organic acid (mM) to the total products (mM) × 100

^a, b, c^Means different superscripts differ significantly (*P* < 0.05)

### Gene expression

**Fig. 2:**
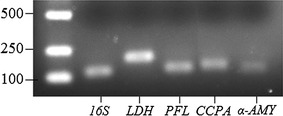
PCR products of genes. The *left line* is a DNA ladder marker (bp)

**Fig. 3:**
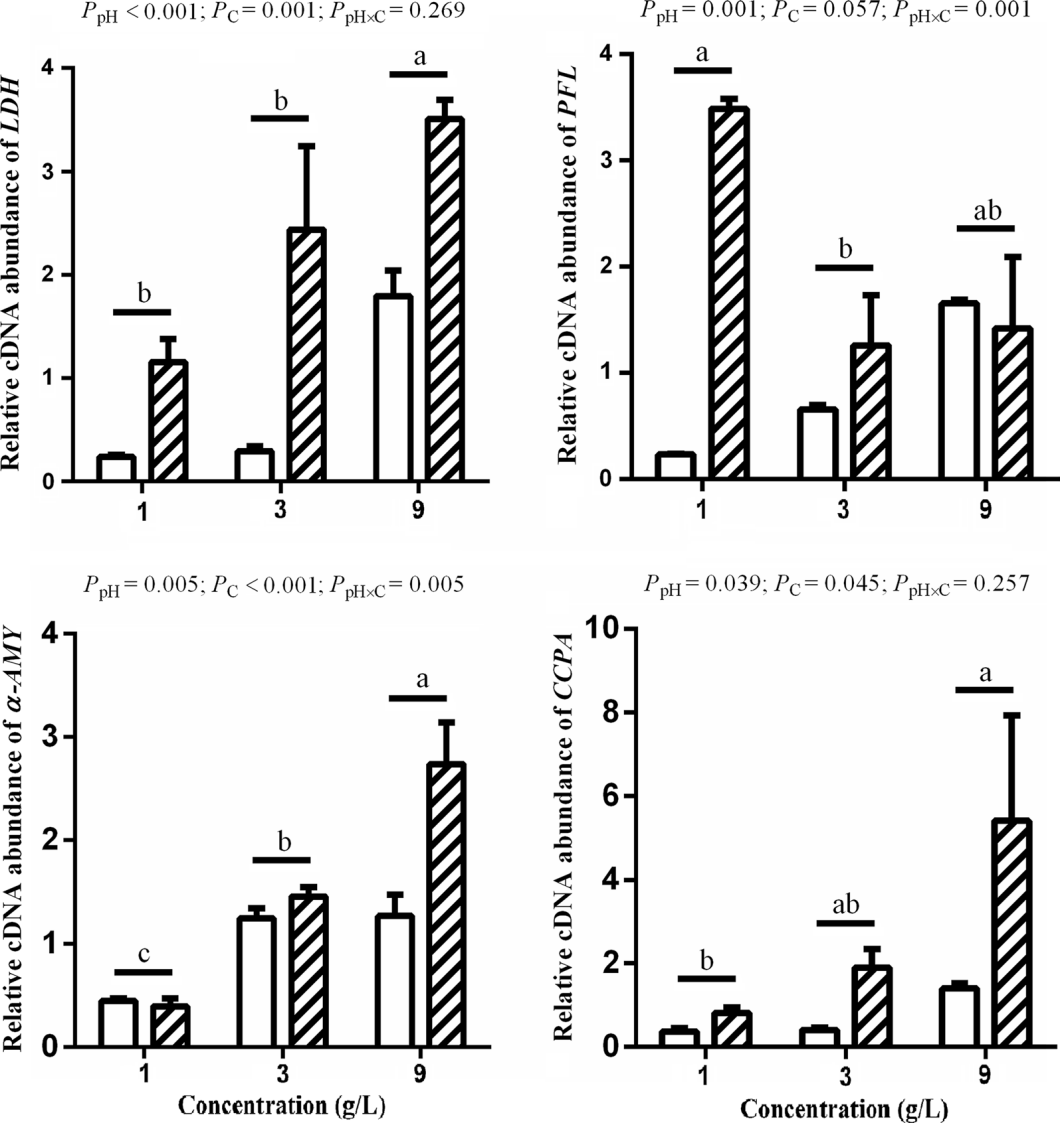
Effects of soluble starch concentrations and pH on *LDH, PFL, α*-*AMY* and *CCPA* gene expressions. Soluble starch concentrations are 1 g/L, 3 g/L and 9 g/L;* Columns*  and  represent the pH at 6.5 and 5.5; Values are means (n = 3) with their standard errors represented by *vertical bars*. Significant differences are indicated among three starch concentrations with different superscripts (*a*,* b*, *c*) (*P* < 0.05). The expression of four genes at pH 5.5 were significantly higher than pH 6.5 (*P* < 0.05)

The gel electrophoresis of gene PCR products is shown in Fig. 2. Results confirmed that bands of PCR products were specific for the target genes and suitable for further examination. The results of the relative expressions of *LDH*, *PFL* (gene encoding pyruvate formate lyase), *CCPA* (gene encoding global catabolite control protein A) and *α*-*AMY* genes are shown in Fig. 3. The relative expressions of *LDH*, *PFL*, *CCPA* and *α*-*AMY* genes at pH 5.5 were all higher than those at pH 6.5 (*P* < 0.05). The relative expressions of *LDH* and *CCPA* genes at 9 g/L starch level were higher than those at 1 g/L starch level (*P* < 0.05), and no difference was found between 1 g/L and 3 g/L starch levels (*P* > 0.05). The relative expression of *α*-*AMY* gene was up-regulated in response to the starch levels (*P* < 0.05). The relative expression of *PFL* gene at 3 g/L starch level was lower than that at 1 g/L starch level (*P* < 0.05), but there was no difference between 1 g/L and 9 g/L starch levels (*P* > 0.05).

### Enzyme activity and FDP concentration

The results of the LDH and α-AMY activity and FDP concentration are shown in Table 4. The activity of LDH and α-AMY at pH 5.5 was higher than pH 6.5 (*P* < 0.05). The activity of LDH and α-AMY at 9 g/L starch was higher than those at 1 g/L and 3 g/L starch levels (*P* < 0.05), but no difference was found between 1 g/L and 3 g/L starch levels (*P* > 0.05). The FDP concentration increased with the starch level in a dose-dependent manner (*P* < 0.05), and was higher at pH 6.5 than pH 5.5 (*P* < 0.05).

**Table 4:** Effects of soluble starch concentrations and pH on the specific activity of LDH and α-AMY, and FDP concentrations

Items	PH	Concentration (g/L)			Mean_pH_	SEM_Total_	*P*_pH_	*P*_c_	*P*_pH×c_
		1	3	9					
LDH (U/L)^1^	6.5	1.09	2.71	3.83	2.54^b^	0.906	<0.001	0.007	0.550
	5.5	4.60	5.87	8.90	6.46^a^				
	Mean_C_	2.85^b^	4.29^b^	6.37^a^					
α-AMY (U/L)^1^	6.5	38.52	52.81	59.74	50.36^b^	3.610	<0.001	<0.001	0.027
	5.5	59.90	59.26	88.47	69.21^a^				
	Mean_C_	49.21^b^	56.04^b^	74.10^a^					
FDP (mM/L)^2^	6.5	24.92	30.68	33.38	29.66^a^	0.832	0.032	<0.001	0.371
	5.5	23.29	27.81	32.94	28.01^b^				
	Mean_C_	24.11^c^	29.24^b^	33.16^a^					

^1^Activity (U/L) per 10^7^ cells break down in 1 mL medium

^2^Concentration (mM/L) per 10^7^ cells break down in 1 mL medium

^a, b, c^Means different superscripts differ significantly (*P* < 0.05)

## Discussion

Rumen acidosis is usually defined as consistent decreases in ruminal pH lasting for several hours each day, and the pH below the normal condition has significant impacts on microbial composition and fermentation (Nagaraja and Lechtenberg 2007; Hook et al. 2011). The change of ruminal pH affects most bacterial organic acid fermentations, including *S. bovis* (Silva and Yang 1995; Asanuma and Hino 2000). In general, the pH affects not only the growth of bacteria and the fermentation rate, but also the final product yield. Changing the medium pH may also induce a metabolic shift (Zhu and Yang 2004). The importance of pH in the media, related to readily fermentable substrate levels on the development of acidosis, as being dictated by an accumulation of lactate is supported by our results from the binary linear regression analysis in the present study. The analysis clearly showed that the effects of the pH in the media were more profound than the soluble starch concentration, in terms of the lactate production and FDP concentration, being evidenced by the greater regression coefficient values (absolute values in Table 5) of pH as compared to the values for starch.

**Table 5:** Binary linear regression analysis of lactate production, enzyme activity, FDP concentration and gene expression

Items	Explanatory variable	${\text{R}}_{{_{\text{adjust}} }}^{{^{ 2} }}$	*B*	SME_*b*_	*P* values	
					*P*_R_^2^	*P*_*b*_
Lactate	pH	0.910	0.403	0.141	<0.001	0.012
	C		0.268	0.021		<0.001
LDH	pH	0.725	−1.367	0.247	<0.001	<0.001
	C		0.146	0.036		0.001
α-AMY	pH	0.781	−1.167	0.220	<0.001	<0.001
	C		0.191	0.032		<0.001
FDP	pH	0.765	0.400	0.229	<0.001	0.101
	C		0.248	0.034		<0.001
*LDH*	pH	0.765	−1.215	0.228	<0.001	<0.001
	C		0.181	0.034		<0.001
*PFL*	pH	0.189	−1.035	0.425	0.082	0.028
	C		−0.008	0.062		0.905
*α*-*AMY*	pH	0.593	−0.641	0.301	<0.001	0.050
	C		0.209	0.044		<0.001
*CCPA*	pH	0.401	−0.843	0.365	0.008	0.036
	C		0.152	0.054		0.013

The regression equations are as follows:

Y_Lactate_ = −3.576 + 0.403X_pH_ + 0.268X_C_

Y_LDH_ = 7.57 − 1.367X_pH_ + 0.146X_C_

Y_α-AMY_ = 6.171 − 1.167X_pH_ +0.191X_C_

Y_FDP_ = –3.471 + 0.4X_pH_ + 0.248X_C_

Y_*LDH*_ = 6.505 − 1.215X_pH_ + 0.181X_C_

Y_*PFL*_ = 6.241 − 1.035X_pH_ − 0.008X_C_

Y_*α*-*AMY*_ = 2.944 − 0.641X_pH_ + 0.209X_C_

Y_*CCPA*_ = 4.398 − 0.843X_pH_ + 0.152X_C_

The activity of LDH and *LDH* abundance at pH 5.5 was higher than pH 6.5 in the present study, and the findings are consistent with the results of Asanuma et al. (Asanuma et al. 1999). However, the final concentration of lactate and the lactate percentage to the total organic acids were significantly higher at pH 6.5 than pH 5.5. Although the activity of α-AMY and *α*-*AMY* abundance at pH 5.5 were also higher than pH 6.5, the concentration of FDP (a glycolytic intermediate) at pH 5.5 remained lower than pH 6.5, suggesting that a low flow rate of soluble starch fermentation at pH 5.5. At the same time, the cell growth at pH 6.5 was faster than at pH 5.5, providing the direct evidence that soluble starch was fermented at a higher rate at pH 6.5. resulting in more lactate produced.

In contrast an in vivo study showed a small amount of lactate was detected in the early stage of rumen acidosis (Nagaraja and Lechtenberg 2007). This could be explained by the tolerance of the bacteria to low pH. In normal conditions (pH > 6.5), most of the rumen bacteria grow well, and lactate produced by *S. bovis* and *Lactobacilli* will rapidly be metabolized by lactate utilizers (such as *Selenomonas ruminantium* and *Megasphaera elsdenii*) (Nocek 1997). With an imbalance between the acid production and the acid utilization, the accumulation of organic acids in the rumen makes rumen pH decline (pH < 5.5). The low pH could result in many Gram negative bacteria disappear, including lactate-utilizing bacteria such as *Megasphaera elsdenii* and *Selenomonas ruminantium*, because they are sensitive to low pH (Hernández et al. 2014). Conversely, there is an increase in the population of some Gram positive bacteria, especially *S. bovis*, known as a prominent lactate producing bacteria, leading to lactate accumulation in the rumen which provokes a metabolic acidosis (Russell and Hino 1985; Nocek 1997).

It seems that the amount of metabolic substrate plays a more important role in breaking down the balance among the bacterial production and utilization, and ruminal absorption of the acids. An increase of readily fermentable carbohydrates in diet initially leads to an increased growth rate of most rumen bacteria (Hook et al. 2011). This is due to the increased substrate available for oxidation by the various groups of bacteria and less competition for the substrates among them. As fermentation proceeds and the concentration of products of fermentation increases, the absorptive capacity of the rumen papillae could be capped, and the concentration of VFA and lactate in the rumen increases, leading to a decrease of rumen pH.

Additionally, Asanuma et al. found when excess glucose in the media led to more lactate produced by *S. bovis*, whereas the formate formation did not change (Asanuma et al. 1999), suggesting glucose abundance would lead to a fermentation pattern shift. Since *S. bovis* can produce α-amylase, which hydrolyses starch to single sugars that are further converted to pyruvate for organic acid fermentation (Walker 1965). In this work, with the increase of soluble starch concentration, the activities of LDH and α-AMY, as well as the abundance of *LDH* and *α*-*AMY,* were all increased. In addition, a *CCPA* gene that encodes global catabolite control protein A (CCPA), which is involved in the transcriptional regulation of LDH and PFL, was up-regulated with excess soluble starch supplied. These results suggest that the abundance of soluble starch regulates the fermentation pathways is at the transcriptional level by CCPA in *S. bovis* (Asanuma et al. 2004; Antunes et al. 2012). Moreover, LDH of *S. bovis* specifically requires FDP for activity (Wolin 1964), so LDH activity would be enhanced greatly with an increase of soluble starch concentration. These may all contribute to the increment of lactate fermentation. Once lactate produced is greater than that utilized, the balance breaks, lactate accumulation occurs and pH drops. From this point of view, readily fermentable substrate is the root cause of the decline in rumen pH. We also calculated the total organic acid production per gram of the soluble starch consumed, and found that the lactate production per gram of the soluble starch consumed tended to decrease with the increase of soluble starch supplementation. In other amylolytic lactate bacterial reported so far, the lactate production yield per unit substrate was higher at a low starch concentration than that at a high starch concentration (Mercier et al. 1992; Yumoto and Ikeda 1995; Reddy et al. 2008). But, the mechanism for this phenomenon remains to be clarified.

In summary, the results obtained in the current study demonstrate that *S. bovis* S1 produced lactate at both pH 6.5 and pH 5.5, and the lactate production was dependent on the substrate starch concentration, though the production rate (lactate yield per unit of starch consumed) changed in an opposite way. These were regulated at the transcription level in response to both pH and substrate concentration. In addition, the lactate production in *S. bovis* S1 was more sensitive to the pH changes than to the starch level.

## Abbreviations

LDH: lactate dehydrogenase
PFL: pyruvate formate-lyase
α-AMY: α-amylase
FDP: fructose-1,6-diphosphate
CCPA: global catabolite control protein A
VFA: volatile fatty acids
NFC: non-structural carbohydrate
OD: optical density
CFU: colony forming units
